# Archaeological Support for the Three-Stage Expansion of Modern Humans across Northeastern Eurasia and into the Americas

**DOI:** 10.1371/journal.pone.0012472

**Published:** 2010-08-30

**Authors:** Marcus J. Hamilton, Briggs Buchanan

**Affiliations:** 1 Department of Biology, University of New Mexico, Albuquerque, New Mexico, United States of America; 2 Department of Anthropology, University of New Mexico, Albuquerque, New Mexico, United States of America; 3 Santa Fe Institute, Santa Fe, New Mexico, United States of America; 4 Department of Archaeology, Simon Fraser University, Burnaby, Canada; 5 Department of Anthropology, University of Missouri, Columbia, Missouri, United States of America; University of Utah, United States of America

## Abstract

**Background:**

Understanding the dynamics of the human range expansion across northeastern Eurasia during the late Pleistocene is central to establishing empirical temporal constraints on the colonization of the Americas [1]. Opinions vary widely on how and when the Americas were colonized, with advocates supporting either a pre-[2] or post-[1], [3], [4], [5], [6] last glacial maximum (LGM) colonization, via either a land bridge across Beringia [3], [4], [5], a sea-faring Pacific Rim coastal route [1], [3], a trans-Arctic route [4], or a trans-Atlantic oceanic route [5]. Here we analyze a large sample of radiocarbon dates from the northeast Eurasian Upper Paleolithic to identify the origin of this expansion, and estimate the velocity of colonization wave as it moved across northern Eurasia and into the Americas.

**Methodology/Principal Findings:**

We use diffusion models [6], [7] to quantify these dynamics. Our results show the expansion originated in the Altai region of southern Siberia ∼46kBP , and from there expanded across northern Eurasia at an average velocity of 0.16 km per year. However, the movement of the colonizing wave was not continuous but underwent three distinct phases: 1) an initial expansion from 47-32k calBP; 2) a hiatus from ∼32-16k calBP, and 3) a second expansion after the LGM ∼16k calBP. These results provide archaeological support for the recently proposed three-stage model of the colonization of the Americas [8], [9]. Our results falsify the hypothesis of a pre-LGM terrestrial colonization of the Americas and we discuss the importance of these empirical results in the light of alternative models.

**Conclusions/Significance:**

Our results demonstrate that the radiocarbon record of Upper Paleolithic northeastern Eurasia supports a post-LGM terrestrial colonization of the Americas falsifying the proposed pre-LGM terrestrial colonization of the Americas. We show that this expansion was not a simple process, but proceeded in three phases, consistent with genetic data, largely in response to the variable climatic conditions of late Pleistocene northeast Eurasia. Further, the constraints imposed by the spatiotemporal gradient in the empirical radiocarbon record across this entire region suggests that North America cannot have been colonized much before the existing Clovis radiocarbon record suggests.

## Introduction

Anatomically modern humans expanded out of Africa ∼50–60kBP [10], and by ∼45kBP had reached as far east as southeast Asia [11], Australia [12] and southern Siberia [13]. Over the following 30,000 years or so, modern humans expanded their biogeographic range across northeast Eurasia colonizing the mainland Far East, the Japanese archipelago, Beringia, and the Americas. Within this broad framework, however, much of the spatial and temporal dynamics of this vast range expansion are still very much in question.

The colonization of the Americas is a particularly contentious issue [2], [14], [15]. Researchers are divided into several camps, with some contending that the Clovis archaeological complex represents the initial human colonists of the Americas, and generally support a terrestrial colonization pathway across Beringia, through an ice-free corridor between the Laurentide and Cordilleran ice sheets and onto the northern Plains sometime after ∼14k calBP [6], [14], [15]. Others support a pre-Clovis occupation of the Americas with several alternative scenarios, including either a pre-last glacial maximum (LGM) [2] or post-LGM [1], [14] colonization, following colonization pathways either by land or by a sea-faring coastal colonization along the Pacific Rim. Other proposed alternatives include a trans-Atlantic colonization of the Americas from Europe via the east coast of North America [5] and a trans-Arctic pre-LGM colonization via Arctic Canada [4]. As such, just about every conceivable route into the late Pleistocene Americas via land or sea are currently supported by one research team or another. However, the archaeological and biological evidence supporting these alternatives varies widely.

Whatever the exact timing and whichever route or routes of colonization were taken, the Americas were colonized as part of the broader biogeographic expansion of modern humans across the planet. Abundant genetic data demonstrate that northeast Eurasia was the genetic homeland of the native peoples of the Americas [16], [17], [18], more specifically the region between the Altai Mountains of southern Siberia and the Amur Basin/Okhotsk region of the Eurasian Far East [1], [16]. Genetic data also demonstrate that the first human colonists of the Korean Peninsula [17] and the Japanese archipelago [18] were also of northeast Eurasian descent, suggesting that the entire Late Pleistocene population of this vast region encompassing northeastern Eurasia and the Americas, ultimately originated from an initial population expansion out of southern Siberia ∼45k calBP. While genetic data are somewhat equivocal on the divergence times of Native Americans from their Eurasian ancestors due to the extreme sensitivity of estimated evolutionary rates [19], [20], [21], [22], [23], [24], [25], such data do provide archaeologically testable hypotheses concerning the geographic location and timing of prehistoric expansion events.

Thus, at a broad scale, the colonization of the Americas is best understood within the broader context of population expansions and movements within northeastern Eurasia over the late Pleistocene. While most researchers agree on the general timing and location of the initial southern Siberian expansion, and its subsequent expansion to the northeast [26], [27], finer-grained details about population movements over the late Pleistocene are less clear. In particular, there is ongoing debate about whether or not southern Siberia was depopulated over the height of the LGM [28], [29]. Other researchers have identified a “Beringian pause” in the colonization of the Americas, where populations in greater Beringia were geographically isolated from the rest of northeast Eurasian populations long enough for specific genetic differences to accumulate, which were then brought to the Americas by the first colonizers [30], [31]. Recently, these ideas were incorporated into a three-stage colonization model of the Americas proposed by Kitchen and colleagues [8], [9] where they identify 1) an initial expansion phase ∼40k calBP (latter stages of MIS 3) in southern Siberia, at which time northeast Eurasians became genetically differentiated from other Eurasians; 2) a long Beringian pause, where populations in greater Beringia became isolated from other populations for most of MIS 2, from ∼32-16k cal BP; and 3) a second post-LGM expansion phase starting ∼16k calBP, which led to the colonization of eastern Beringia, and subsequently the rest of the Americas.

Here we use diffusion models [6], [7] to analyze a large dataset of Upper Paleolithic radiocarbon dates from northeast Eurasia to quantify the spatial dynamics of this population expansion. In particular, we use spatiotemporal gradients in the radiocarbon record of Upper Paleolithic northeastern Siberia to identify the location and number of expansion events, and discuss the implications of our results in terms of the various colonization models. We show that the internal dynamics of these Upper Paleolithic expansions place clear empirical constraints on the timing of the initial colonization of the Americas, and we suggest the kinds of spatiotemporal patterns that would have to emerge from future research in order to support certain early colonization scenarios.

## Methods

### The diffusion model

Diffusion analyses have been particularly successful in quantifying prehistoric population expansions [e.g.], [ 6], [7], [32,33] as they recover underlying statistical patterns in datasets, rather than relying on dates from single sites, which may or may not be statistical outliers [see 10]. As such, the benefits of diffusion analyses are that they identify overall trends in datasets (such as gradients in earliest occupations), as well as place individual sites within the broader statistical context of regional archaeological records, and so are statistically powerful.

The mathematical details of the different types of diffusion analyses appropriate in archaeology are covered in detail elsewhere [for recent reviews see 34], [35]. Here we use a simple reaction-diffusion model that combines a logistic population growth term with a diffusion term, which describes the spread of the population in two spatial dimensions. The resulting equation is termed the Fisher equation:
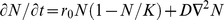
(1)where 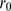 is the intrinsic annual population growth rate (∼4%, or 0.04 in humans), *N* is population size or density, *K* is the carrying capacity of the local environment, *D* is the diffusion coefficient (in km yr^−1^), and 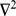 is the Laplacian operator describing the diffusion of the population, *N*, in two dimensions. Equation 1 produces traveling waves of colonists, radiating out in concentric circles from an initial point of origin. The velocity, *v*, of this wave front is given by
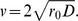
(2)Under this simple model the velocity of expansion given by equation 2 is constant.

### Statistical theory

We estimate the velocity of a population expansion (equation 2) statistically by quantifying the slope of the relation between the absolute distance travelled by the expanding population, over the time taken to travel that distance. We use a procedure similar to ref [6], which was based on statistical procedures developed in previous work [7], [32], [33]. First, we identify a point of origin for the population expansion, which is assumed to be approximated spatially by the location of the earliest radiocarbon-dated site in the data set. Second, for each site in the data set we measure the distance from the site to the point of origin in km using great circle arc distances. Third, we produce a bivariate plot of calibrated dates and distances, and fourth, fit regression models to the upper bound of this relation, thus estimating the rate of change in distance with respect to the change in time, i.e., the velocity of the population expansion.

The bivariate plots of calibrated dates by distance produced roughly triangular shaped plots. Following previous research [6], [7], [32], [33] radiocarbon dates appear on the *y*-axis and distances on the *x*-axis because there is significantly more error in the date estimates than the distance estimates, which are measured, essentially, without error. We are interested in quantifying the upper boundary of these plots as this boundary identifies the earliest recorded site for a given distance as the inverse slope of this relation provides an estimate of the expansion velocity (+/−error), i.e., 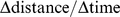. We utilize a commonly-used binning method where each site in the database is sorted into bins of a constant width based on its distance from the point of origin. Within each bin the earliest calibrated dates are then extracted, thus providing estimates of the earliest occupations for a given distance from the origin.

We use two regression techniques to estimate the velocity of this slope. First, we use an ordinary least squares (OLS) bisector model. In an OLS bisector model two OLS regression slopes are calculated from the two functions; 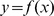 and 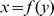, which in our case is 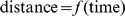 and 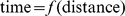. The estimated velocity in the first function is the slope 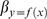 of 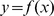. The velocity from the second function is the inverse slope,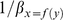, of 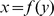. The estimated velocity of the population expansion is then the average of the two estimated velocities. The strength of the OLS bisector model is that the overall slope is calculated from two models representing the two bounding functions, where the first model assumes all measurement error occurs on the *y*-axis and the second assumes that all measurement error occurs on the *x*-axis. The second technique we apply is a reduced major axis (RMA) regression model, which assumes measurement error is equally divided along the *y* and *x* axes. Maps were produced in Google Earth Pro, and statistical analyses were performed in *R* (www.r-project.org/).

### Data

**Figure 1:**
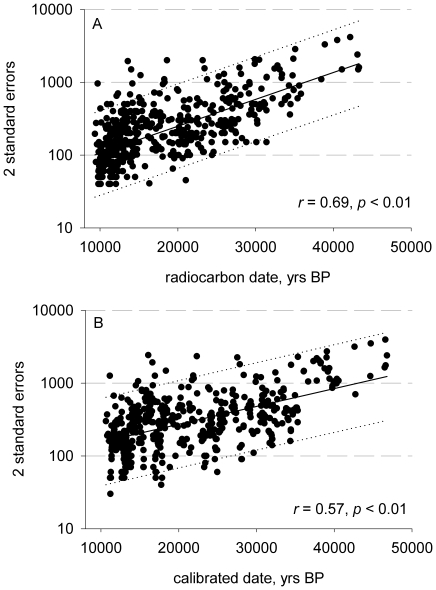
Bivariate plots of calibration errors as a function of occupation dates. A) Uncalibrated dates and B) calibrated dates. Solid lines are exponential fits and dotted lines are 95% prediction intervals of the model. The error rates of a date are exponentially related to the age of that date. Thus an arbitrary error cut off rate of 1000, for example, would exclude all radiocarbon dates older than 40,000 years old. Further, while the oldest dates have the largest absolute errors, they vary at the same multiplicative rate as younger dates. In fact the older dates are less variable than the younger dates: all dates older than about 30,000 years BP (on either plot) fall within the 95% predictions of the exponential fit.

We first compiled a dataset of radiocarbon-dated Upper Paleolithic archaeological sites from northeastern Eurasia and Beringia above 45°N [following 26], including sites from Siberia, northern China and Alaska. Data were compiled from multiple sources for a total sample size of 516 individual radiocarbon dates from 143 sites representing 257 occupational site-phases ranging in age from ∼46.6kBP to ∼12kBP (see Dataset S1). The dataset we use in this paper consists of the earliest dated occupation event at each of the 143 sites. We attempted to include all available published radiocarbon dates, though some omissions are unavoidable. From our original data, we excluded dates where we had information that they were derived from surface finds, or otherwise unreliable contexts. The only sites we excluded based on large error ranges were those identified as clear statistical outliers from the overall data set. We did not exclude sites based on an arbitrary error range because in a data set that spans over 30,000 years for both uncalibrated and calibrated dates there is a clear exponential relation between the estimated occupation and the associated errors, such that older sites have exponentially more error (Figure 1).

All dates were calibrated with the downloadable version of CalPal using the CalPal-2007_HULU_ calibration curve [36], [37], [38]. We used pooled mean dates for site-phases with multiple radiocarbon assays. When stratigraphic or site component information was available we used this as the criterion in which to calculate pooled mean dates. In cases where no stratigraphic or component information was available we used CalPal to calculate probability distributions from the uncalibrated dates (see Analyses S1). We pooled uncalibrated dates that occurred within non-overlapping distributions (see supporting information). We calculated pooled mean dates using Calib 5.1 [39].

## Results

**Figure 2:**
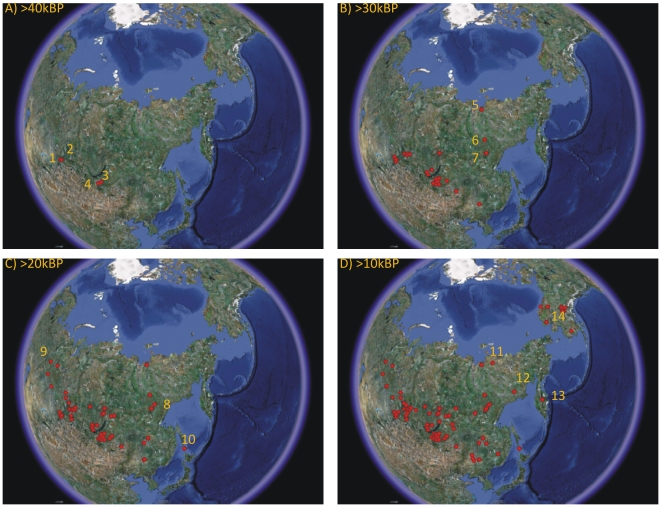
Distribution maps of the expansion of radiocarbon-dated archaeological sites across northeastern Eurasia above 45°N in 10-thousand year increments. A) The earliest sites are located along the southern Siberia-northern Mongolia border, including Kara-Bom (1), the earliest site in the database, Kara-Tenesh (2), Kandabaevo (3), and Podzvonkaya (4). B) By ∼30k BP sites are found along the far-western border of Beringia following a north-south line, including Yana RHS (5), Ikhine 2 (6), and Ust-Mil 2 (7). C) There are no new sites in western Beringia over the next 10-thousand years, except Ezhantsy (8), which appears along the previously defined western Beringian border. However, sites appear to the northwest, such as Rychkovo (9), and further south, including Ogonki 5 (10) on Sakhalin Island. D) After the LGM sites rapidly appear along a temporal gradient across the greater Beringian region, including Berelekh (11), Siberdik (12), and Ushki (13), reaching eastern Beringia (Alaska) by ∼13.8k BP, Swan Point (14).

Distribution maps (Figures 2A–D) show that the four earliest sites (Kara-Bom, Kara-Tenesh, Kandabaevo, and Podzvonskaya) predating 40k calBP are located in southern Siberia. Over the next ten thousand years, the latter stages of MIS 3, sites appear throughout northeast Eurasia, reaching the far-western boundary of Beringia by about 30k calBP. Below 45°N, the Eurasian Far East, including both the Korean Peninsula and the Japanese archipelago were colonized by at least ∼35k calBP [40], [41], [42], [43], [44], [45], [46]. From ∼30-20k calBP, over MIS 2, including the LGM, the expansion front does not expand any farther to the east, though Ogonki 5 on Sakahlin Island, is dated to ∼23k calBP, and is the earliest dated site to the immediate north of the Japanese archipelago. After the LGM there is a rapid expansion across central Beringia and the earliest sites in eastern Beringia (Alaska) date to ∼13.7k calBP.

### Diffusion analysis

The oldest site in the dataset is Kara-Bom at 46,620+/−1,750 cal BP, and so is used to represent the point of origin for the population expansion. The bivariate plot of the data of calibrated date by distance yielded a roughly triangular distribution with a reasonably well-defined linear upper bound representing the gradient of earliest occupations of the population expansion radiating out of southern Siberia.

The OLS bisector model through the binned data produced the following slopes; 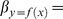
0.15 and 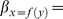
5.97, and so a velocity of 1/5.97 = 0.17 (OLS regression: *r* ^2^ = 0.85, *p*<0.001). The overall estimated velocity is then 0.16 (0.14–0.21 CIs) km per year (OLS bisector regression: 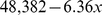, *r* ^2^ = 0.85, *p*<0.001). The slope estimated using an RMA regression through the binned data was identical to the OLS bisector estimate, yielding an estimated velocity of 0.16 (0.13–0.19 CIs) km per year (RMA regression: 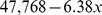, *r* ^2^ = 0.88, *p*<0.001). Thus we conclude that the average velocity of the expansion of modern humans across northeastern Eurasia over the entire expansion phase was about 0.16 km per year.

**Figure 3:**
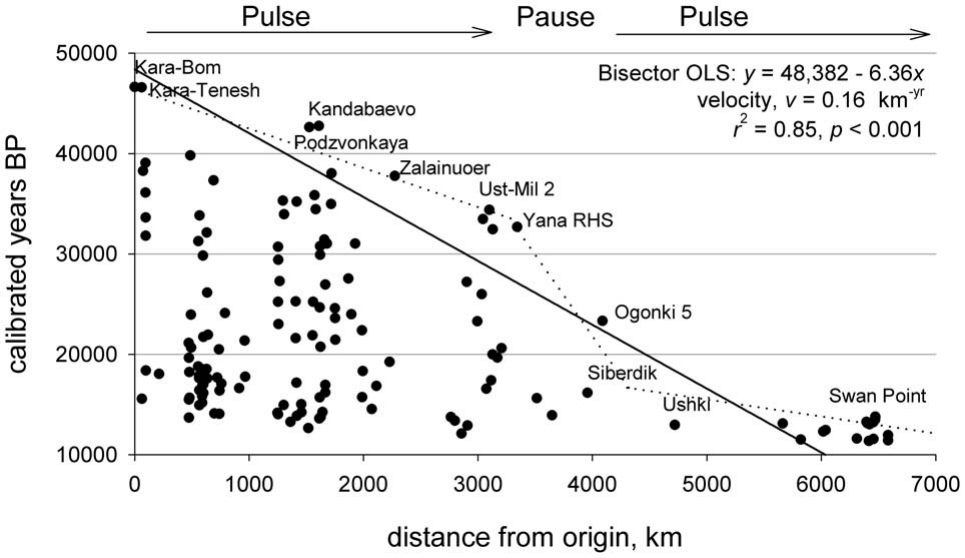
Bivariate plots and regression models of calibrated dates and distance from origin for each site. A) The solid line is a bisector OLS regression model (see Methods for details) through the earliest occupations per 500 km bin. Results demonstrate that over the ∼35,000 year expansion period the wave front traveled at an average velocity of about 0.16 km per year. The dotted line shows that the actual wave front seems to be three-stage with an initial pulse (∼46-32k BP), followed by a long pause spanning the LGM (∼32-17k BP), followed by a second pulse after the LGM (∼17-14k BP; see text for details).

However, both the distribution maps in Figure 2 and the bivariate plots in Figure 3 indicate that the population expansion was not a continuous process. Indeed, there was a initial expansion from ∼46-32k calBP (during the latter stages of MIS 3), followed by a hiatus from about ∼32-16k calBP (onset and maximum of MIS 2) along the far-western border of Beringia, which was then followed by a second expansion after ∼16k calBP (late MIS 2) following the LGM. Estimates of the first expansion suggest a velocity of ∼0.25 km per year (∼3,300 km over ∼14,000 years) during the MIS 3 interstadial, while the second expansion was considerably faster at ∼1 km per year during late MIS 2 (∼2,400 km over ∼2,500 years). These archaeological dynamics suggest a three-stage pulse-pause-pulse expansion process, similar to the three-stage colonization model recently proposed by Kitchen and colleagues [8], [9] based on genetic data.

## Discussion

Through the combined use of distribution maps and diffusion analysis our data suggest that the expansion of modern humans across northeast Eurasia played out in three stages. These stages included an initial expansion from ∼47k-32k BP, from southern Siberia to western Beringia during the later stages of the last interstadial (MIS 3), a long expansion hiatus from ∼32k-16k BP spanning the onset and maximum of the last glacial (MIS 2), followed by a second expansion after ∼16k BP into eastern Beringia (Alaska) during the later stages of MIS 2 as the climate warmed rapidly after the LGM.

The three-stage process we demonstrate using radiocarbon dates is remarkably similar to the three-stage model proposed by Kitchen and colleagues [8], [9]. It is particularly interesting that the long population hiatus from ∼32-17k calBP suggested by their results [25], [26] is very similar to the hiatus identified in the radiocarbon record. The geographic location of this hiatus is unclear from the genetic data, but they suggest that it occurred within greater Beringia, and the lack of an associated archaeological record may be due to the formation of the Bering Strait, the submergence of the Bering landmass, and the lack of archaeological research in far northeastern Siberia [8], [9]. However, our data suggest such isolation may have occurred further to the west along the western border of Beringia, albeit during the same time period. Whether or not southern Siberia underwent complete depopulation over the LGM is a matter of debate, though there seems to be archaeological evidence that at least some settlement persisted throughout MIS 2 [29]. Because we only analyze the earliest occupations of multi-component sites the data we present here do not directly address this question, but if southern Siberia was not entirely depopulation over this period, evidence of a long hiatus lends circumstantial archaeological support for an isolated population of late Pleistocene hunter-gatherers in the correct general region over the predicted time period.

Estimates of the average velocity of the population expansion over the entire expansion phase are slow, at an average ∼0.16 km per year. This expansion velocity is considerably slower than other known late Pleistocene population expansions, including the initial modern human colonization of western Europe, 0.4 km per year [47], the re-colonization of northern Europe, 0.8 km per year [7], and the Clovis expansion in North America, 7.6 km per year [6]. The slow pace of the northeastern Eurasian expansion was likely due, in some degree, to the harsh, dry, and cold climates of northern Eurasia during most of the Pleistocene. Indeed, the colonization of northern latitudes required a series technological innovations, including tailored clothing, shelters, abundant fuel for fires, and specialized technologies to support life in extreme environments [48], [49], [50]. However, the early colonization of northern latitudes of European Eurasia ∼40k BP [51], [52] indicates that these technological innovations developed rapidly in the few thousand years following the expansion of modern humans out of Africa ∼50k BP [49].

The Yana RHS site in northwestern Siberia dated to ∼32k BP [53] indicates that hunter-gatherer populations had the ability to live above the arctic circle during the later stages of the relatively warm MIS 3, though the following expansion hiatus over much of MIS 2 suggests that such populations could not compete with the dry and cold climate of the onset and maximum of the last glacial in Beringia. It should be noted however that while Yana RHS is recognized as the earliest evidence of the initial colonization of arctic Siberia, Figure 2B shows that the occupation at Yana RHS falls almost directly on the wave front of the initial population expansion from ∼46k BP – 32k BP, suggesting that the northern latitudes of Siberia were colonized as part of the general population expansion across northeast Eurasia.

The initial population expansion from ∼46k BP-32k BP occurred during the last interstadial, MIS 3, and was likely facilitated by relatively warm and moist climatic conditions, which saw a mixture of boreal forests and parklands throughout much of southern Siberia and western Beringia, and increasingly arid conditions toward Beringia, similar to the present climate [54], [55]. Indeed, this period of expansion coincides closely with the MIS 3 climatic optimum (∼39k-33k BP [55]), though the estimated velocity of the expansion was slow, ∼0.25 km per year. With the onset of the last glaciation, MIS 2, in central Siberia and western Beringia mean annual temperatures fell by up to 4°C, and annual precipitation dropped dramatically [54], [55]. During the LGM regional temperatures fell by up to 10°C, and rainfall by ∼250 mm followed by intense loess deposition and the expansion of arctic tundra, indicative of cold hyper-arid conditions [54]. However, the hyper-aridity of MIS 2 meant that glaciation in northeast Eurasia was extremely limited [56], [57], [58], as opposed to the extensive glaciations in North America at similar latitudes over this time period. Indeed, these climatic conditions must have been among the most extreme faced by hunter-gatherers over our evolutionary history. While it is entirely feasible that Paleolithic groups did not completely depopulate the region over the LGM, population densities must have been extremely low and localized, providing the ecological and cultural conditions for the long period of isolation hypothesized by Kitchen and colleagues [8], [9], Bonatto and Salzano [31], and Tamm and colleagues [30].

After the LGM the climate warmed rapidly and boreal forests were re-established throughout much of the region by ∼14k BP [54] while a seemingly unique central Beringian landscape of mixed shrubland-grassland supported a diversity of late Pleistocene megafauna [59]. As post-LGM conditions were much more conducive to human foraging, population expansion proceeded rapidly [60] at a velocity of ∼1 km per year across Beringia (Figure 2D), about 4-times faster than the pre-LGM expansion, with the earliest evidence of human colonization of eastern Beringia at Swan Point, Alaska, shortly after 14k BP. Further expansion to the south was temporarily halted by the Cordilleran and Laurentide glaciers. Current evidence places the earliest Paleoindian populations (i.e., Clovis) below the ice sheets on the northern Plains of North America by at least ∼13.4k BP [6], [15], [61], shortly after the appearance of the ice-free corridor linking southeastern Beringia to the northern Plains of North America along the eastern boundary of the Rockies ∼14k BP [62]. However, it is likely that Clovis populations reached the northern Plains sometime earlier than current archaeological evidence suggests, but due to a combination of a small founding population [9], [63] at extremely low densities on the landscape, and the taphonomic and depositional conditions of the northern Plains over the last 14,000 years, the archaeological visibility of the very initial phases of this colonization would be very low.

We now consider the evidence for various alternative colonization models in light of the empirical record of the northeastern Eurasian Upper Paleolithic, and the gradients identified above.

### Alternative models

**Figure 4:**
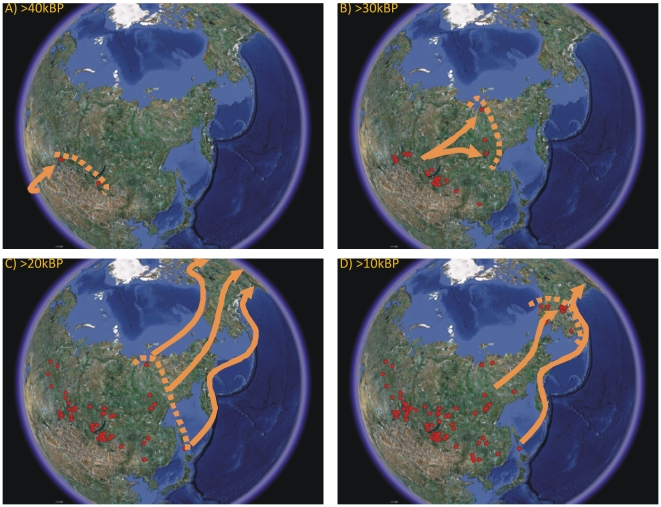
Proposed alternative timings and trajectories of colonization routes into the Americas. Dashed lines are approximate boundaries of the extent of settlement based on dated archaeological sites, and solid arrows are hypothesized population movements. A) The initial expansion into southern Siberia from central Asia is relatively uncontroversial. B) Similarly, the expansion from southern Siberia to far-western Beringia by ∼30k calBP is uncontroversial. C) Three proposed pre-LGM colonization routes include coastal, terrestrial, and trans-Arctic routes, however, currently there are no archaeological sites beyond western Beringia to support these routes. D) Post-LGM models include the traditional trans-Beringian route, and a coastal route. The trans-Beringian route is the best supported by current archaeological data, and there is no archaeological evidence to suggest the coastal route was a major factor in colonization, albeit complicated by Holocene sea level rise along the Pacific Rim. However, it is entirely feasible that as colonists expanded across the Beringian mainland local groups close to the southern coast may have included aquatic resources in the diet. However, there is no evidence of full maritime cultures anywhere along the Pacific Rim until well into the Holocene.

Four alternative models for the colonization of the Americas have been proposed: 1) a pre-LGM terrestrial expansion; 2) a pre-LGM Pacific rim coastal expansion; and 3) a pre-LGM trans-Arctic expansion; and 4) a post-LGM/pre-14k calBP coastal colonization model (Figures 4A–D). We considered each of these models in turn.

Figure 4 shows the distribution of dated archaeological sites in the study region in 10,000 year increments, the dashed lines are the approximate eastern boundaries of human occupation suggested by the distribution of ages from radiocarbon-dated archaeological occupations, and the bold arrows are suggested expansion trajectories. Figures 4A and B are relatively uncontroversial, as most researchers would agree that the earliest occupations occur in southern Siberia, most likely from central Asian populations between ∼50k-45k BP, and expand to include the Japanese archipelago by ∼35k BP and the western border of Beringia by ∼30k BP.

**Figure 5:**
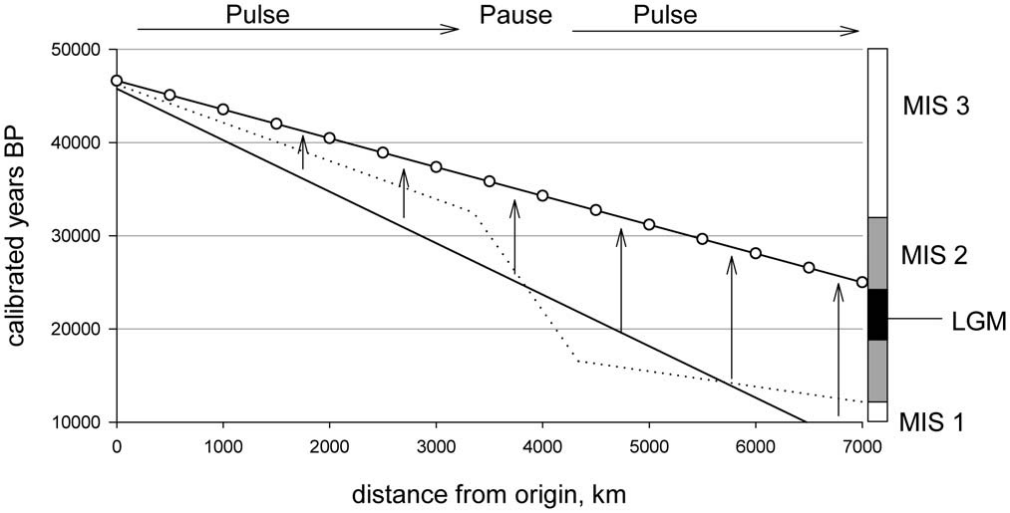
Schematic of the empirical wave front fit (solid line), the three-stage expansion model (dotted lines), and a hypothetical pre-LGM colonization model (solid line with closed circles). The schematic illustrates the minimum radiocarbon gradient that the Eurasian record would have to show in order to support a pre-LGM colonization of the Americas. The velocity of expansion would have to be more than twice as fast as the empirical gradient in figure 3 (∼0.4 vs 0.16 km^−yr^), and sites throughout Beringia would have to be at least twice as old as the current archaeological record indicates.

Figure 4C, which spans the onset and maximum of the last glacial (MIS 2), illustrates the three proposed pre-LGM colonization models proposed in the literature, which involve colonization pathways either along the northern or southern coasts of Beringia, or across the Beringian mainland. However, the distribution of sites, and the boundaries outlined in figure 4C and 4D demonstrate that currently there is no archaeological evidence of human settlements to the east of the extreme western border of Beringia until well after the LGM, ∼16k calBP. Moreover, Figure 5 provides an estimate for the minimum gradient that the Eurasian radiocarbon record would have to demonstrate in order to support a pre-LGM colonization of the Americas. Assuming the initial expansion out of southern Siberia occurred ∼45k calBP a pre-LGM colonization of the Americas would require a continuous expansion process with no pause along the western boundary of Beringia. The velocity of this expansion would have to be twice as fast as the empirical gradient shown in panel A and sites throughout Beringia would have to be at least twice as old as the current archaeological record indicates. A pre-LGM colonization would also require the extensive human occupation of Beringia during the extreme cold, hyper-arid conditions of much of MIS 2. Although there are only a handful of dated Upper Paleolithic sites throughout western and central Beringia, a pre-LGM colonization of the Americas would require a radical reformulation of the Eurasian Upper Paleolithic archaeological record as it currently stands, and all new dates would have to deviate from the current pattern in a highly systematic way.

Figure 4 also demonstrates that while a pre-Clovis coastal colonization of the Americas has undergone a recent resurgence in support due to the radiocarbon dating of Monte Verde II, Chile, [64] currently there is no archaeological evidence from the Eurasian record to support this model. While Ogonki 5 (Sakhalin Island) is the oldest dated archaeological occupation north of Japan, at 23,310 calBP, currently there are no known coastal Upper Paleolithic sites anywhere along the Pacific Rim north and east of Sakahlin Island before ∼13k calBP, at Ushki on the Kamchatka Peninsula. Nor are there any pre-14k calBP sites along the Pacific coast of the Americas to suggest that colonization followed this route during this period. Of course, any evidence for a coastal colonization is severely hampered by sea level rise over the Holocene along the steep continental shelf of the Pacific Rim, which would have either been destroyed, or at least obscured any archaeological evidence along the immediate coastline [3]. However, the lack of evidence of any occupations during this critical phase (let alone Upper Paleolithic maritime cultures) anywhere along the ∼20,000 km length of the Pacific Rim from Hokkaido to Tierra del Fuego should not simply be ignored or explained away.

### Conclusions

Our results demonstrate that the human expansion across northeastern Eurasia over the late Pleistocene followed a three-stage pulse-pause-pulse dynamic, with the pulse phases corresponding with the relatively warm phases of late MIS 3 and post-LGM MIS 2, and the pause corresponding with the harsh glacial conditions of early- to mid-MIS 2. The radiocarbon record of Upper Paleolithic northeastern Eurasia provides no support for the pre-LGM colonization of Beringia beyond its far-western border, or the Americas. Indeed, for humans to have colonized the Americas much before Clovis (as currently dated [6], [65]) would require major changes to the northeast Eurasian Upper Paleolithic archaeological record, including a much faster colonization velocity, no expansion hiatus, and a Beringian archaeological record more than twice as old as current evidence suggests. Indeed, the currently available radiocarbon data place robust temporal constraints on the colonization process across this entire region, and are well-explained by a relatively simple three-stage diffusion similar to the model proposed by Kitchen and colleagues [8].

## Supporting Information

Dataset S1 3 worksheets containing the data set for this project. The first worksheet contains all individual radiocarbon dates included in the analysis. The second worksheet contains estimated dates for individual occupations at each site. The third worksheet contains the earliest dated occupation at each site. This is our primary database.
(0.36 MB XLS)

Analyses S1 The file contains individual worksheets of calibration and clustering output for all multicomponent sites where we did not have stratigraphic information for individual dates. The clustering on the y-axis allowed us to identify individual occupation events, which were then pooled to estimate the date of the occupation event.
(8.78 MB XLS)
